# Metabolic Flux Analysis during the Exponential Growth Phase of *Saccharomyces cerevisiae* in Wine Fermentations

**DOI:** 10.1371/journal.pone.0071909

**Published:** 2013-08-13

**Authors:** Manuel Quirós, Rubén Martínez-Moreno, Joan Albiol, Pilar Morales, Felícitas Vázquez-Lima, Antonio Barreiro-Vázquez, Pau Ferrer, Ramon Gonzalez

**Affiliations:** 1 Instituto de Ciencias de la Vid y del Vino (Consejo Superior de Investigaciones Científicas, Universidad de la Rioja, Gobierno de La Rioja), Logroño, Spain; 2 Department of Chemical Engineering, Universitat Autònoma de Barcelona, Barcelona, Spain; Texas A&M University, United States of America

## Abstract

As a consequence of the increase in global average temperature, grapes with the adequate phenolic and aromatic maturity tend to be overripe by the time of harvest, resulting in increased sugar concentrations and imbalanced C/N ratios in fermenting musts. This fact sets obvious additional hurdles in the challenge of obtaining wines with reduced alcohols levels, a new trend in consumer demands. It would therefore be interesting to understand *Saccharomyces cerevisiae* physiology during the fermentation of must with these altered characteristics. The present study aims to determine the distribution of metabolic fluxes during the yeast exponential growth phase, when both carbon and nitrogen sources are in excess, using continuous cultures. Two different sugar concentrations were studied under two different winemaking temperature conditions. Although consumption and production rates for key metabolites were severely affected by the different experimental conditions studied, the general distribution of fluxes in central carbon metabolism was basically conserved in all cases. It was also observed that temperature and sugar concentration exerted a higher effect on the pentose phosphate pathway and glycerol formation than on glycolysis and ethanol production. Additionally, nitrogen uptake, both quantitatively and qualitatively, was strongly influenced by environmental conditions. This work provides the most complete stoichiometric model used for Metabolic Flux Analysis of *S. cerevisiae* in wine fermentations employed so far, including the synthesis and release of relevant aroma compounds and could be used in the design of optimal nitrogen supplementation of wine fermentations.

## Introduction

Over the past few decades, the intensified emissions of greenhouse gases and aerosol particles mainly derived from industrial activity and transport have led to one of the major challenges in the history of mankind: global warming [1]. Apart from known consequences such as the melting of the polar ice caps, this phenomenon is also affecting agriculture. In fact, the increase in temperature has already had a noteworthy effect on, among others, the grape and wine industry [2].

Global warming has been shown to cause lower yields in *Vitis vinifera*, changes in plagues and microbiological diseases and drastic modifications in grape physiology. With respect to the latter, changes in acidity, phenolic maturation, tannin content and sugar concentration have been proven to occur, especially in warm climates [3]. Among these parameters, increased sugar concentrations may cause important changes in the physiology of *Saccharomyces cerevisiae*, the yeast species mainly responsible for the alcoholic fermentation that takes place during winemaking. High sugar concentrations induce an osmotic stress response in yeast, which can result in stuck or sluggish fermentations and lead to increased formation of fermentation by-products such as glycerol and acetic acid [4,5].

Although the physiology of *S. cerevisiae* has been thoroughly studied and characterized over the last century due to the status of this yeast as model organism, the distribution of metabolic fluxes occurring during wine fermentations has not been tackled in depth. Due to the intrinsic dynamic nature of this process, yeast metabolism undergoes a series of physiological adaptive changes in response to the continuous environmental variations that take place, clearly hampering its understanding.

During the last decade, several laboratories have aimed to study yeast physiology under winemaking conditions. Varela et al. [6] described the metabolic flux distribution of yeast during sluggish and normal fermentations using batch cultures. This group has further extended this approach by developing a genome-scale dynamic flux balance model that allows prediction of metabolite production in batch cultures [7]. In this context, Clement et al. [8] studied the fermentation process in a “multistage bioreactor system”, where two or four fermenters, operating in continuous mode, were connected in tandem mimicking sequential stages of standard batch wine fermentations. This allowed the achievement of a series of metabolic steady states resembling those found in each stage of wine fermentation. Nevertheless, this cultivation strategy was not used for further Metabolic Flux Analysis (MFA) studies. As an alternative, we have recently developed a simple approach for metabolic phenotyping of yeast during wine fermentations. In short, batch fermentations are first systematically characterized, including measurements of growth and consumption/production rates of the main metabolites along the whole process. Based on the evolution of physiological parameters, the fermentation is divided into three defined phases: phase I, corresponding to the yeast exponential growth phase; phase II, when nitrogen becomes a limiting nutrient; and phase III, where most of the ethanol is produced at near zero growth rate. Finally, based on the batch parameters, specific feeds are defined in order to independently mimic each of these phases using continuous cultures [9]. This strategy allows for an easy and reliable calculation of the distribution of metabolic fluxes [10].

In the present study, we investigated the impact of growth temperature and sugar content on the distribution of metabolic fluxes of *S. cerevisiae* EC1118 growing under phase I conditions at fermentation temperatures typical for red (28 °C) and white (16 °C) wines. Emulating an extreme increase in sugar concentration motivated by global warming, we have used a synthetic must with 280 g L^-1^ glucose and compared the obtained results with a control must containing 240 g L^-1^ glucose. A control must with 240 g L^-1^ glucose and another, emulating an extreme increase in sugar concentration motivated by global warming with 280 g L^-1^ glucose, have been used. In all cases, media were supplemented with potassium metabisulphite in order to mimic cellar conditions. During this first phase, yeast reaches its maximum growth rate (μ_max_) that depends on fermentation temperature, organic and inorganic nitrogen sources are available and not growth-limiting, and high sugar and low ethanol concentrations are present in the fermenting must.

The proposed and validated model includes reactions from central carbon and nitrogen metabolism. With regard to previously used models, the latter has been expanded to incorporate transport reactions for ammonium and 19 different amino acids, their anabolism and/or catabolism and, for the first time, pathways involved in the synthesis of aroma precursors. Moreover, the present work combines the application of a stoichiometric model for analyzing the metabolic flux distribution of *S. cerevisiae* under winemaking conditions using continuous cultures in bioreactors.

## Material and Methods

### Strains and culture conditions

*Saccharomyces cerevisiae* LALVIN EC1118, the strain used in this study, is a commercial wine yeast strain isolated from the Champagne region (France) produced and commercialized by Lallemand Inc. (Ontario, Canada). The strain was grown at 28 °C and routinely maintained at 4 °C on YPD plates containing 2% glucose (w/v), 2% peptone, 1% yeast extract and 2% agar, and in glycerol stocks at -80 ^°^C.

### Batch and continuous cultivations

A modification of a chemically defined synthetic must previously described [11] was used both for the batch and the continuous cultures. The medium contained the following components (expressed per litre): glucose, 240 or 280 g; malic acid, 6 g; citric acid, 6 g; Difco^TM^ Yeast Nitrogen Base w/o amino acids and ammonium sulphate (BD, Sparks, USA), 1,7 g; ammonium chloride, 306 mg; alanine, 97 mg; arginine, 245 mg; aspartic acid, 29 mg; cysteine, 14 mg; glutamic acid, 80 mg; glutamine, 333 mg; glycine, 12 mg; histidine, 23 mg; isoleucine, 22 mg; leucine, 32 mg; lysine, 11 mg; methionine, 21 mg; phenylalanine, 25 mg; proline, 400 mg; serine, 52 mg; threonine, 50 mg; tryptophan, 116 mg; tyrosine, 13 mg; valine, 29 mg; ergosterol, 15 mg; sodium oleate, 5 mg; Tween 80, 0.5 mL and potassium metabisulphite, 60 mg. All cultures were run in a DASGIP parallel fermentation platform (DASGIP AG, Jülich, Germany) equipped with four SR0400SS vessels. Batch cultures were performed in duplicate using 300 mL as the initial working volume and were used to establish the specific production and consumption rates of the main metabolites (e.g. glucose, glycerol, ethanol, CO_2_, biomass, acetic acid, lactic acid and succinic acid) throughout the whole fermentative process. Continuous cultures were performed in duplicate at a constant volume of 200 mL at near μ_max_ dilution rate (D): 0.25 h^-1^ for those fermentations performed at 28 ^°^C and 0.1 h^-1^ for those performed at 16 °C. These D values had been experimentally determined previously in the aforementioned batch cultures. Agitation was maintained at 250 rpm and the temperature kept at 28 or 16 ^°^C using a water bath and a 1:1 water/ethylenglycol-cooled chiller. During batch and continuous cultures, the pH of the medium was kept at 3.5 by the automated addition of 2N NaOH. Anaerobic conditions were maintained by gassing the headspace of the bioreactors with pure nitrogen gas (3.5 sL h^-1^). The use of spargers was avoided in order to reduce ethanol stripping. The effluent fermentation gas measured every 30 s for determination of CO_2_ concentration with a GA4 gas analyzer (DASGIP AG). For the inoculation of both batch and continuous cultures, EC1118 was grown in Falcon tubes containing 25 mL YPD and incubated at 28 °C and 150 rpm orbital shaking for 48h in order to reach stationary phase. Cells were then washed twice in sterile deionized water, resuspended in 5 mL of the synthetic must and inoculated to an initial OD_600_ 0.2. Steady states were sampled only after all continuous cultures had been running for at least five residence times and the CO_2_ production rate, the biomass values and the concentration of the main metabolites were constant.

### Analysis of extracellular metabolites

#### HPLC quantification of main metabolites

The concentration of glucose, glycerol, ethanol and lactic, acetic and succinic acid was determined following the protocol described by Quirós et al. [12] using a Surveyor Plus chromatograph (Thermo, Fisher Scientific, Waltham, MA) equipped with a refraction index and a photodiode array detector (Surveyor RI Plus and Surveyor PDA Plus, respectively) on a 300 × 7.7 mm HyperREZ^TM^ XP Carbohydrate H+ (8 µm particle size) column and guard (Thermo, Fisher Scientific). The column was maintained at 50 °C and 1.5 mM H_2_SO_4_ was used as the mobile phase at a flow rate of 0.6 mL min^-1^. Prior to injection in duplicate, samples were filtered through 0.22 µm pore size nylon filters (Symta, Madrid, Spain) and diluted when necessary 10 or 20-fold.

The concentration of each amino acid was analyzed in duplicate according to the method of Gomez-Alonso et al. [13] using an Accela 600 chromatograph (Thermo, Fisher Scientific) equipped with a PDA detector and a 250 × 4.6 mm ACE C18-HL ID (5 µm particle size) column and guard (ACE, Aberdeen, Scotland).

The concentration of ammonium was determined enzymatically using the R-Biopharm assay kit Cat. No. 11 112 732 035 (Darmstadt, Germany).

#### GC-MS determination

The volatile fraction of steady states was analyzed using headspace solid-phase micro extraction coupled with gas chromatography-mass spectrometry (HS-SPME/GCMS) by a modification of the protocol described by Pozo-Bayon et al. [14]. A Thermo Scientific Trace GC Ultra gas chromatograph equipped with a Thermo Scientific Triplus Autosampler and coupled to a Thermo Scientific ISQ mass detector was used for gas chromatography-mass-spectrometry.

Five fusel alcohols (propanol, isoamyl alcohol, amyl alcohol, isobutanol and 2-phenylethanol) were quantified using 2 ml of fermentation broth. One gram of sodium chloride was added to the sample placed in a 20 mL headspace vial, followed by 10 µL of 2000 ppm internal standard solution (4-methyl-2-pentanol and 1-nonanol in 0.5% ethanol). Briefly, the vial was tightly capped with a PTFE/Silicone cap and then heated for 10 min at 70 °C. Then, a Supelco 100 µm PDMS fiber was exposed to the headspace of the sample vials for 30 min and desorbed in the GC inlet for 4 min. The instrument was fitted with a 30 m × 0.25 mm TG-WAXMS A fused-silica capillary column, 0.25 mm film thickness (Thermo, Fisher Scientific). The GC temperature program was as follows: 40 °C (5 min hold), 3 °C min^-1^ up to 200 ^°^C and 15 ^°^C min^-1^ up to 240 ^°^C (10 min hold), while the 0.75 mm I.D. SPME liner was held at 180 ^°^C. Helium was used as the carrier gas at a flow rate of 1 mL min^-1^, operating in split mode (ratio 30). For the MS detector, the temperatures of transfer line and ion source were both 250 °C, ionization mode was electron impact at a voltage of 70 eV and acquisitions were performed in SIM mode (dwell time 50 ms). Instrument control, data analysis and quantification results were carried out with Xcalibur 2.1 software. Volatile compounds were identified and quantified by comparison with standards, and analyses were carried out in duplicate.

### Biomass composition analyses

#### Determination of cell growth and biomass dry weight

To determine cell growth during the course of batch fermentations, the optical density (OD) was monitored using a Shimadzu UV-1800 Spectrophotometer (Shimadzu, Europe GmbH, Duisberg, Germany). When necessary, samples were diluted with deionized water to obtain OD_600_ measurements in the linear range of 0.1-0.4 units. OD data were then transformed to dry cell weight values using a calibration curve for *S. cerevisiae* EC1118 in the synthetic must described above. When steady states were reached, biomass dry weight was determined in triplicate by filtering 5 ml (for fermentations run at 28 °C) or 15 mL (for fermentations run at 16 °C) of the cultures followed by 10 mL distilled water through a 25 mm, 0.45 µm pore size pre-dried and pre-weighed nitrocellulose filter (Millipore, Billerica, USA). Filters were then heat dried in an oven at 70 °C until weight was constant (12-24 h).

#### Biomass lyophilization

Samples of cultivation broth from all steady states were centrifuged at 10,000 rpm for 5 min and the cell pellet was washed three times in sterile distilled water. The recovered pellet was immediately frozen by immersion in liquid nitrogen and lyophilized during 72 h. Once lyophilized, cell pellets were additionally dried in an oven at 65 °C for 24 h.

#### Total carbohydrate content

To determine the biomass total carbohydrate content, an aqueous solution of lyophilized biomass was prepared at a concentration of 0.1 g L^-1^ and subjected in triplicate to the phenol-sulphuric acid method as described by Segarra et al. [15] using a standard curve of glucose (Sigma-Aldrich).

#### Total protein content and amino acid composition

Total protein content and amino acid composition of the biomass was determined following the protocol described by Carnicer et al. [16]. This allowed us to estimate the molar fraction of each amino acid in the average protein.

#### Elementary composition

Biomass recovered from 50 mL of each steady state was washed three times with deionized water, dried in an oven at 70 °C for 72 hours and crushed thoroughly using a porcelain mortar and pestle. The fine powdered biomass was dried again in the same conditions for 24 h and 1 mg analyzed in an EA 1110 CHNS-O elemental analyzer (CE-Instruments/Thermo Fisher Scientific) coupled to an E3200 autosampler and a thermoconductivity detector.

In order to determine the biomass ash content, the powdered biomass was placed in a pre-weighted crucible and calcinated in a muffle furnace at 800 °C for 24 h. The difference in weight was measured with an AB204-S electronic precision scale (Mettler, Toledo, Columbus, USA).

### Consistency check, data reconciliation and statistical analysis

Data obtained during continuous cultures were checked for consistency and reconciled based on a χ^2^-test (p≤0.05) as proposed by Wang and Stephanopoulos [17]. The test proposed by Lange and Heijnen [18] was used for verification of consistency and reconciliation of macromolecular and elementary biomass compositional data. This test allowed for the estimation of the non-measured biomass lipid and nucleic acid content.

The physiological parameters and metabolic flux values obtained in all conditions were compared by means of Student’s t-test at 95% confidence level. Principal Components Analysis (PCA) was performed in order to identify relevant experimental condition effects using IBM SPSS win 19.0 software (IBM Corp., Armonk, USA).

### Stoichiometric model

The stoichiometric model used to represent the metabolic network of *S. cerevisiae* EC1118 was adapted from the model of Varela et al. [6] (see Appendix S1). While the reactions involved in central carbon metabolism have been used with minor modifications, nitrogen metabolism has been thoroughly revised and expanded. Therefore, the uptake reactions for ammonium and the 19 amino acids present in the medium have been included in the model (proline transport was not included as it is not degraded under anaerobic conditions according to Ingledew et al. [19]). On the other hand, reactions involved in either synthesis or catabolism of amino acids have been included according to the following criteria. When the ratio “incorporation rate into the biomass/uptake rate” (mol gDW^-1^ h^-1^/mol gDW^-1^ h^-1^) of a specific amino acid was ≥ 1, the biosynthetic pathway for that amino acid was included. In contrast, when this ratio was < 1, it has been assumed that there is an excess of such compound in the cell and, therefore, the corresponding degradation pathway was included. The total cell content of each amino acid residue was estimated from the molar fraction of each amino acid in the total protein.

Additionally, some alternative amino acid degradation routes, such as the Ehrlich pathway, which involves the synthesis of isoamyl alcohol, active amyl alcohol and isobutanol, among others, have also been included in the proposed stoichiometric model.

The final metabolic network consists of *m*=88 metabolites and *n*=82 reactions (Appendix S1). The existence and stoichiometry of each reaction was verified using the Saccharomyces Genome Database (SGD, www.yeastgenome.org) and the Kyoto Encyclopedia of Genes and Genomes (KEGG, www.genome.jp/kegg/).

### Software

All numerical calculations were performed using Matlab 2010b (MathWorks Inc., Natick, MA, USA). CellDesigner 4.2 (Systems Biology Institute, Tokio, Japan) was used for metabolic network design and Cell-Net-Analyzer 9.4 [20] for MFA.

## Results and Discussion

### Quantitative physiology of *S. cerevisiae* EC1118 during anaerobic continuous cultures at near μ_max_ dilution rate

#### General physiology

Although the metabolism of *S. cerevisiae* has been extensively described and studied under different laboratory growth conditions, data regarding key physiological parameters in the different stages of wine fermentation are not easily found in the literature. One of the main objectives of the present work is to contribute to the understanding of yeast physiology by providing accurate production and consumption rates of the main metabolites during the initial stages of wine fermentation in different enological conditions. Since there are important physiological and transcriptional differences between laboratory and industrial wine yeast strains [21], the broadly used wine yeast strain EC1118 was chosen as a model in this work.

Results for consumption/production rates (Table S1) were experimentally obtained from two independent replicates for each condition of glucose concentration, temperature and dilution rate (see Material and methods) and represent the basis for all the calculations performed in this work. Due to the essential need for rates accuracy in order to achieve correct calculated fluxes, special attention was paid in their determination following the recommendations proposed by Heijnen and Verheijen [22]. All balances (carbon, nitrogen and redox) closed with >95% recovery in all cases.

**Figure 1:**
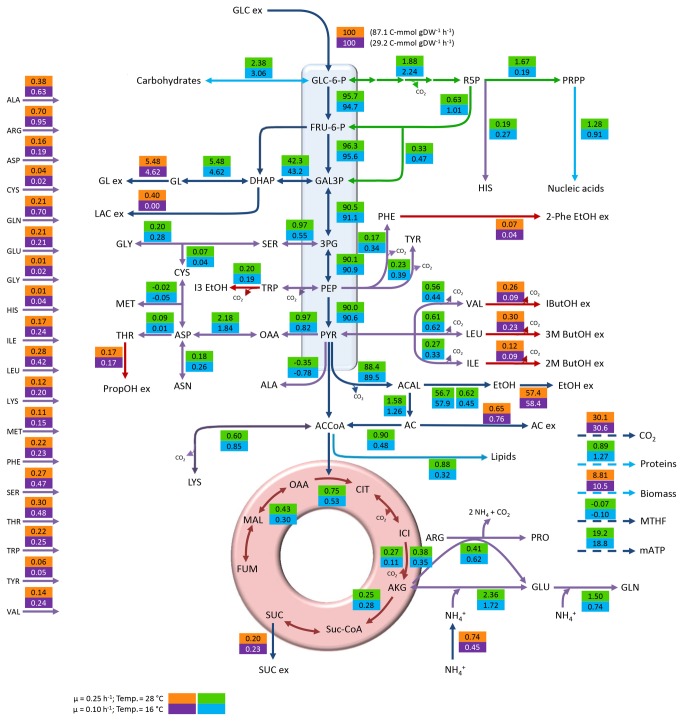
Distribution of metabolic fluxes at 240 g L^-1^ glucose expressed as C-mmol per 100 C-mmol glucose. The measured specific glucose uptake is shown next to the normalized value. Orange/purple boxes: measured and reconciled values; Green/blue boxes: calculated values.

**Figure 2:**
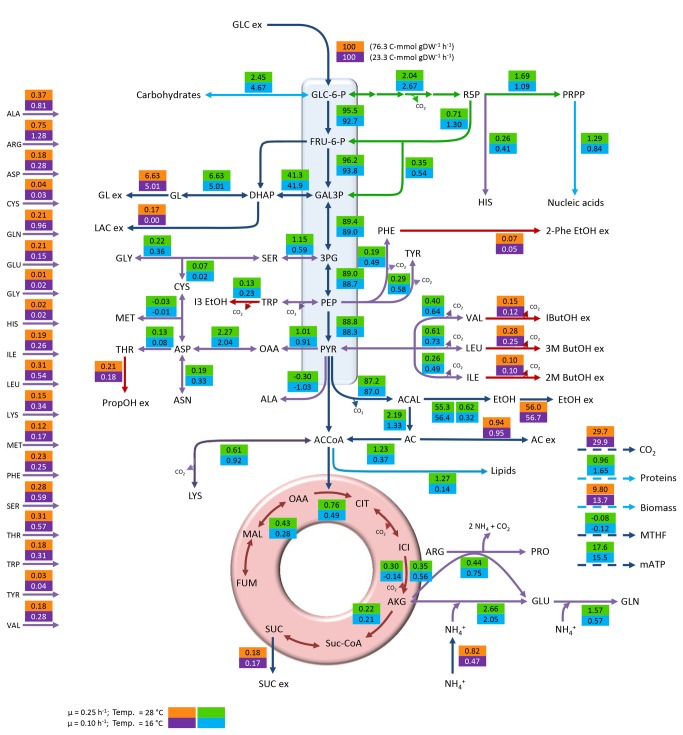
Distribution of metabolic fluxes at 280 g L^-1^ glucose expressed as C-mmol per 100 C-mmol glucose. The measured specific glucose uptake is shown next to the normalized value. Orange/purple boxes: measured and reconciled values; Green/blue boxes: calculated values.

From a general point of view, a strong effect of temperature and dilution rate on the consumption or production rates of the different metabolites could be observed, with higher rates at 28 °C. As an example, glucose uptake rates, representing the main entrance of carbon into the system (carbon skeletons of amino acids also contribute to C uptake) were approximately three times higher at 28 °C and D = 0.25 h^-1^ at both sugar concentrations than at 16 °C and D = 0.1 h^-1^ (87.1 vs. 29.2 C-mmol gDW^-1^ h^-1^ at 240 g L^-1^ and 76.3 vs. 23.3 C-mmol gDW^-1^ h^-1^ at 280 g L^-1^, Figures 1 and 2). These differences must be mainly due to the difference in growth rate and not to the effect of temperature, as exactly the same relationship had been previously observed between *S. cerevisiae* growing at 30 °C in glucose limited chemostats operated at D = 0.3 h^-1^ and D = 0.1 h^-1^ [23]. On the other hand, an impact of sugar concentration was also observed, although this effect was less pronounced (Table S1). Several authors have described that both temperature and growth rate affect physiology of *S. cerevisiae* [21,24,25]. Our results showed that the combination of a decreased temperature and dilution rate always caused the same effect, reducing the consumption or production rate of the different metabolites. However, the maximum specific growth rate (µ) is strongly affected by temperature, which hampers the dissection of the effect due to temperature from that caused by the specific growth rate [24]. In fact, a large overlap between genes reported to be regulated by temperature and genes controlled by growth rate has also been demonstrated [21,24,26]. However, other relevant studies have also shown that the effect of dilution rate on parameters such as glucose consumption, glycerol [23,27], ethanol [23], or biomass production [24] was stronger than the effect of temperature. In our opinion, it would be very interesting to look deeper into the temperature effect since Tai et al. [24] described the slight overlap existing between transcriptomic databases of *S. cerevisiae* wine strain from previous low-temperature adaptation studies [28] and lab strains [29–31]. This fact would be due to the many peculiarities characterizing a grape must such as low pH, high sugar concentration or mixed nitrogen sources present at low concentration.

In all cases of this study, between 81.3 and 84.9% of carbon uptake was used in energy production (measured as ethanol and CO_2_). Sugar concentration exerted a slight (not significant) effect on ethanol and CO_2_ yields on glucose, with yields always being higher at 240 g L^-1^ glucose. On the other hand, the highest acetic acid yields were found at 280 g L^-1^ glucose (Table 1). In contrast, temperature and dilution rate affected biomass and glycerol yields. Higher biomass yields were observed at 16 °C (µ=0.1 h^-1^) while higher glycerol yields were calculated at 28 °C (µ=0.25 h^-1^) (Table 1).

**Table 1:** Yields on glucose (C-mol C-mol^-1^ glucose) for the main metabolites resulting from anaerobic continuous cultures.

	240 g L^-1^ Glucose		280 g L^-1^ Glucose	
	16 °C	28 °C	16 °C	28 °C
Ethanol	0.555 ± 0.005^a^	0.557 ± 0.002^a^	0.532 ± 0.007^a^	0.541 ± 0.009^a^
CO_2_	0.291 ± 0.001^a^	0.292 ± 0.001^a^	0.281 ± 0.005^a^	0.287 ± 0.008^a^
Glycerol	0.044 ± 0.001^a^	0.053 ± 0.001 ^ab^	0.047 ± 0.001^a^	0.064 ± 0.001^b^
Acetic acid	0.007 ± 0.000 ^ab^	0.006 ± 0.000^a^	0.009 ± 0.000^b^	0.009 ± 0.000^b^
Biomass	0.100 ± 0.004 ^ab^	0.086 ± 0.003^a^	0.129 ± 0.002^b^	0.095 ± 0.001^a^
Carbon recovery	0.998 ± 0.006	0.994 ± 0.004	0.998 ± 0.009	0.997 ± 0.012

Different superscripts indicate statistically significant differences between values.

Pathways involved in energy production are strongly regulated, regardless of culture conditions [32,33]. As a result, and as indicated above, not significant differences in ethanol and CO_2_ yields were observed between different growth conditions as previously reported by other authors [23]. On the other hand, glycerol, acetic acid and biomass yields are more related to the internal redox balance [34,35] and therefore presented higher variability depending on growth conditions (Table 1). The distribution of carbon to different metabolites (and throughout the different metabolic pathways) will be more extensively discussed in the following sections.

#### Biomass composition

Although the elemental and macromolecular composition for *S. cerevisiae* biomass has been determined in multiple studies, none of the culture conditions employed resembled the composition of a grape must. We were therefore motivated to perform these analyses in the four conditions studied. Furthermore, the precise knowledge of these values is essential for energetic and metabolic calculations [18]. If the cellular composition changes with culture conditions, it is important to consider the differences in fluxes to the different macromolecular pools [23].

The biomass unit C formulae determined were very similar in the four conditions studied (Table 2) and are consistent with those reported by Albers and co-workers [36]. Nevertheless, significant differences in the N content were found when the formulae obtained were compared to that calculated by Lange and Heijnen [18] for *S. cerevisiae* grown at 0.1 h^-1^ using measurements of dried molecular biomass composition. This finding could be due to differences in the nitrogen sources and concentrations employed in both studies. Albers and co-workers [36] had previously described that growth on different nitrogen sources (ammonium, glutamate and a mixture of twenty amino acids) can alter the amino acidic composition of yeast protein and cause differences in the nitrogen content of the biomass.

**Table 2:** Elemental and macromolecular composition of the biomass in the four conditions used in the present study.

	240 g L^-1^ Glucose		280 g L^-1^ Glucose	
	16 °C	28 °C	16 °C	28 °C
Total proteins (g gDW ^-1^)	0.52 ± 0.07	0.49 ± 0.06	0.51 ± 0.07	0.41 ± 0.06
Total carbohydrates (g gDW ^-1^)	0.30 ± 0.04	0.28 ± 0.03	0.35 ± 0.04	0.26 ± 0.03
Carbohydrates/Proteins (g g^-1^)	0.58 ± 0.1	0.57 ± 0.1	0.69 ± 0.12	0.62 ± 0.12
Ash (g gDW ^-1^)	0.060 ± 0.003	0.060 ± 0.003	0.080 ± 0.004	0.097 ± 0.005
Unit C Formulae^a^	CH_1.604_N_0.209_O_0.488_	CH_1.600_N_0.207_O_0.488_	CH_1.612_N_0.203_O_0.503_	CH_1.612_N_0.201_O_0.471_
Protein C Formulae^b^	CH_1.612_N_0.297_O_0.303_S_0.005_	CH_1.618_N_0.308_O_0.297_S_0.005_	CH_1.614_N_0.307_O_0.296_S_0.005_	CH_1.615_N_0.309_O_0.296_S_0.005_

^a^ Calculated from biomass elemental analysis; ^b^ Calculated from the amino acidic analysis of cell protein. The Unit C Formulae used for lipids, carbohydrate and nucleic acids were taken from Kocková-Kratochvílová [53]. Significant differences were not found between conditions (p < 0.05).

The molar fraction of each amino acid did not significantly vary between the four conditions studied (Table S3), resulting in almost identical protein unit C formulae in all cases (Table 2). Values for this formulae were within ± 10% of those obtained by Lange and Heijnen [18], although significant differences were observed for the sulphur content. Following the aforementioned argument for the biomass unit C formula, these differences could be due to the presence of sulphur amino acids (methionine and cysteine) as part of the nitrogen sources present in the medium employed in the present work.

Proteins were the main constituent of the biomass, normally representing around 50% of the cell dry weight and not showing significant differences between growth conditions. The same trend was also observed for the carbohydrate content, in all conditions close to 30% (Table 2). These two main constituents have been described as growth rate dependent and, while higher amounts of proteins have been quantified at higher growth rates, higher amounts of carbohydrates have been measured at lower growth rates [23,37]. The effect of temperature on these two macromolecular biomass components has also been described. While an increase in total protein content has been measured at the low temperature, increased content in carbohydrates has been determined at the high temperature [21,24]. The absence of statistical differences between conditions could be due to the opposite effect that both parameters (temperature and dilution rate) exert on these macromolecular components. Independently of the conditions, proteins and carbohydrates represent between 67 to 86% of the biomass dry weight. In all cases, higher metabolic fluxes derived towards biomass production resulted in an increase of the metabolic fluxes destined to protein and carbohydrate biosynthesis. Consequently, the nucleic acid and lipid content presented the opposite dynamic (Table 3).

**Table 3:** Fluxes directed towards the synthesis of the main macromolecular components of the biomass expressed as C-mmol per 100 C-mmol glucose.

	240 g L^-1^ Glucose		280 g L^-1^ Glucose	
	16 °C	28 °C	16 °C	28 °C
Carbohydrates	3.06 ± 0.11^b^	2.38 ± 0.07^a^	4.67 ± 0.06^c^	2.45 ± 0.03^a^
Nucleic acids	0.91 ± 0.03^a^	1.28 ± 0.04^b^	0.84 ± 0.01^a^	1.29 ± 0.02^b^
Proteins	1.27 ± 0.04 ^bc^	0.89 ± 0.03^a^	1.65 ± 0.02^c^	0.96 ± 0.01 ^ab^
Lipids	0.32 ± 0.01^a^	0.88 ± 0.03^b^	0.14 ± 0.002^a^	1.28 ± 0.02^c^
Biomass	10.5 ± 0.4^a^	8.8 ± 0.3^a^	13.7 ± 0.2^b^	9.8 ± 0.1^a^

Different superscripts indicate statistically significant differences between values (p < 0.05).

### Metabolic flux analysis

The stoichiometric model used in this work is described in the Materials and methods section. Besides the given details, additional particularities should be noted. Although a grape must is being mimicked, glucose has been used as the only carbon source. A previous characterization of batch cultures using equimolar amounts of glucose and fructose showed that fructose uptake was much lower than glucose consumption during yeast exponential growth phase (data not shown). For this reason, and to improve the accuracy in the determination of sugar uptake, we decided to use glucose as the sole carbon source.

On the other hand, regarding nitrogen metabolism, the ratio “incorporation rate into the biomass/uptake rate” (mol gDW^-1^ h^-1^/mol gDW^-1^ h^-1^) for each amino acid, allowed us to define whether anabolic or catabolic reactions had to be included in each case (see Materials and methods). As shown in Table 4, the ratios for serine, glutamine, threonine and arginine were below 1 in all conditions. Moreover, values for glutamate and tryptophan were below 1 only at 28 °C.

**Table 4:** Ratio “incorporation rate into the biomass/uptake rate” for each amino acid.

	240 g L^-1^ Glucose		280 g L^-1^ Glucose	
	16 °C	28 °C	16 °C	28 °C
Alanine	1.13	0.91	2.15	1.07
Arginine	0.51	0.57	0.70	0.53
Aspartate	1.79	1.36	1.55	1.33
Cysteine	ND	0.90	ND	1.12
Glutamine	0.27	0.23	0.38	0.28
Glutamate	1.29	0.85	2.57	0.96
Glycine	24.12	15.27	47.45	21.44
Histidine	6.57	25.28	17.61	34.99
Isoleucine	1.89	1.68	2.22	1.73
Leucine	1.92	1.73	2.06	1.78
Lysine	3.92	4.23	2.81	3.63
Methionine	1.02	1.08	1.16	1.04
Phenylalanine	2.42	1.50	3.08	1.62
Serine	0.73	0.79	0.85	0.84
Threonine	0.82	0.83	0.89	0.89
Tryptophan	1.11	0.63	2.28	0.73
Tyrosine	42.69	4.24	19.40	8.02
Valine	2.42	2.39	3.06	2.08

ND: Not determined (cysteine consumption was not detected at low temperature).

In order to deepen our knowledge and understanding of yeast amino acid metabolism under winemaking conditions, the model used to represent the metabolic network of *S. cerevisiae* was expanded from that of Varela and co-workers [6] including the transport reactions for 19 amino acids and ammonium and anabolic or catabolic pathways according to the criteria mentioned above. Moreover, according to the current trend in oenological research [38], the metabolism of fusel alcohols (directly related to amino acid breakdown) has also been included.

Under the anaerobic conditions imposed to mimic the environment predominating in wine fermentations, carbon flux was mainly directed to energy production (measured as ethanol and CO_2_) in all cases. The flux towards the tri-carboxylic acid cycle (TCA) remained at levels below 1%, which should be enough to maintain the intracellular levels of key metabolic intermediates [39]. This carbon distribution under anaerobic conditions in a Crabtree positive strain has been previously described [32,33] also under winemaking conditions [6,40]. Figures 1 and 2 show the general flux distribution obtained for each condition expressed as C-mmol gDW^-1^ h^-1^ and normalized to the specific glucose uptake. The reproducibility of the experimental set-up was tested and confirmed using PCA (Figure S1). Metabolic flux distribution will be described and discussed in the following subsections in order to provide an exhaustive analysis of our results. The analysis of the effect of the studied variables on central carbon metabolism has been focused on the glucose 6-phosphate branch point and the trioses phosphate and pyruvate nodes.

#### Glucose 6-phosphate branch point

**Figure 3:**
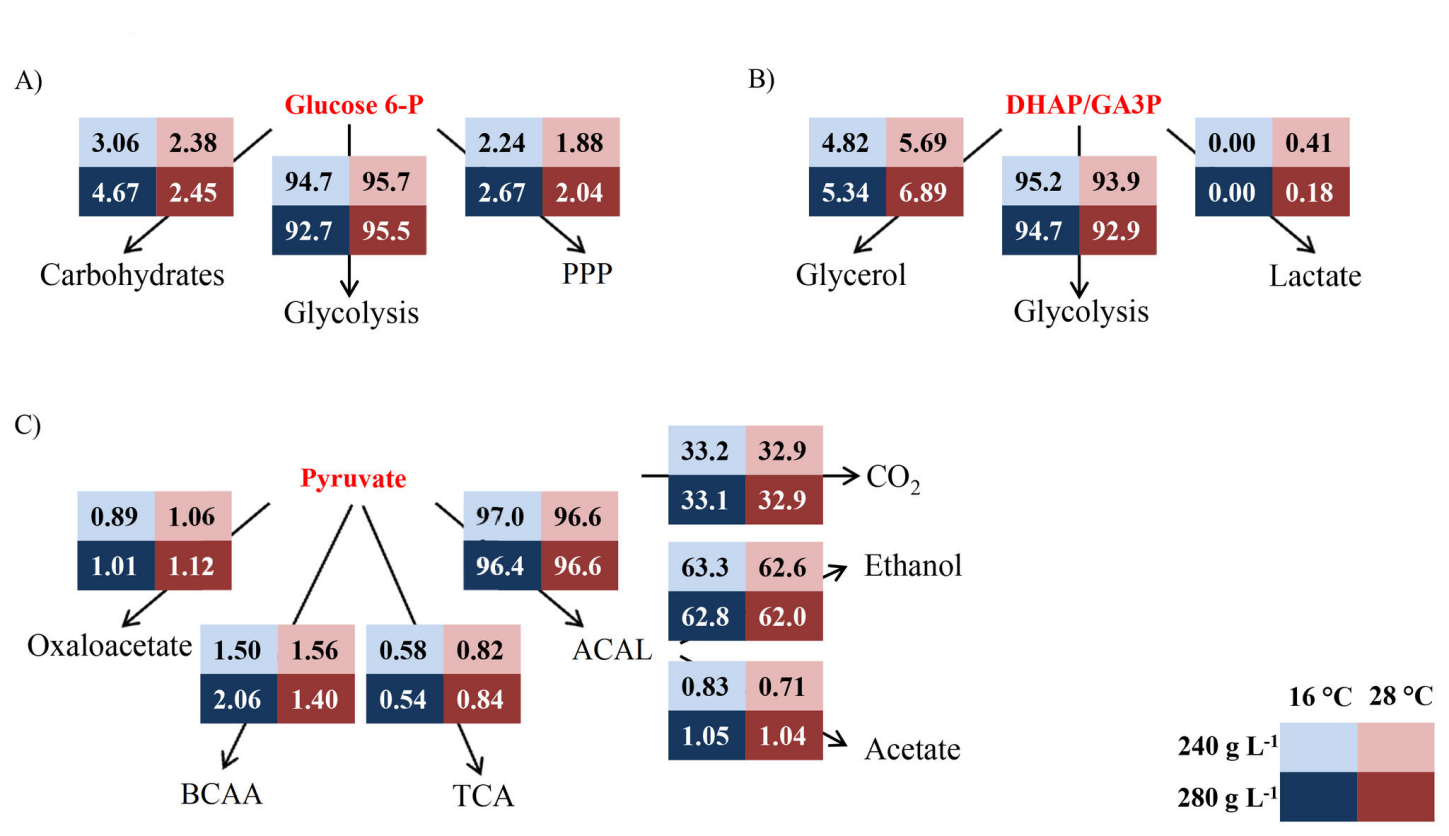
Flux distribution at different nodes. A) Glucose 6-P branch point; B) Trioses phosphate node; C) Pyruvate branch point. PPP: Pentose phosphate pathway; DHAP: Di-hydroxy-acetone phosphate; GA3P: Glyceraldehyde 3-phosphate; BCAA: Brain-chain amino acids; TCA: Tri-carboxylic acid cycle; ACAL: Acetaldehyde. Boxes in light colours indicate 240 g of g L^-1^ glucose. Boxes in dark colours indicate 280 g L^-1^ glucose. Blue indicates 16 °C. Red indicates 28 °C.

The C flux directed to glycolysis was higher at 28 °C than at 16 °C. This resulted in a lower C flux to the two minor branches, i.e., pentose phosphate pathway (PPP) and carbohydrate biosynthesis. On the other hand, the flux directed towards glycolysis at 240 g L^-1^ glucose was higher than at 280 g L^-1^ while those directed to carbohydrate biosynthesis and PPP were higher at 280 g L^-1^; these results are illustrated in Figure 3, panel A. The PPP provides substrates for the synthesis of key products in cellular metabolism. This pathway is involved in the synthesis of nucleic acids (RNA and DNA), some amino acids and the redox carrier NADPH [39], and has been described as growth rate-dependent by different authors [6,23]. Our results showed higher fluxes at the lowest dilution rate used but this fact must be explained by the effect of temperature. Unfortunately, to our knowledge, the effect of temperature on the flux diverted to PPP has not been studied in detail. Nevertheless, as NADPH is mainly consumed in biomass synthesis, higher biomass yields on glucose would demand an increased flux towards the PPP, which, in our study occurs at 16 °C at both sugar concentrations. When the carbon fluxes towards PPP in our culture conditions were analyzed together with those reported by Cadiere et al. [40], Nissen et al. [23] and Varela et al. [6], a wide range of values could be observed. This fact could indicate that the glucose 6-phosphate node is flexible and the flux could be easily modulated depending on cell requirements, at least under anaerobic conditions.

The flux in the remaining branch of this node is directed towards carbohydrate biosynthesis. Following the same trend observed for the PPP, higher fluxes were calculated at 16 °C compared to 28 °C and at 280 g L^-1^ glucose compared to 240 g L^-1^.

As our work focuses on exponentially growing cells and despite the osmotic pressure imposed by high sugar concentrations, the carbon flux directed towards this branch is expected to be destined to structural (mainly glucans and mannoproteins) rather than to reserve carbohydrates (glycogen and trehalose) [6,21,41], as recently observed for *Pichia* *pastoris* [16] and *S. cerevisiae* [18]. Higher fluxes directed towards biomass synthesis were also observed at 16 °C compared to 28 °C and at 280 g L^-1^ of glucose compared to 240 g L^-1^, which would therefore require a higher demand for structural components.

#### Trioses phosphate node

Carbon fate at this branch point was mainly glycolysis in all conditions. However, slight differences were observed depending on the conditions studied. In this way, the glycolytic flux (always accounting for more than 93%) was higher at 16 °C than at 28 °C while the flux towards glycerol was higher at 28 °C. Regarding the sugar concentration effect, the glycolytic flux was higher at 240 g L^-1^ glucose while that directed to glycerol biosynthesis was higher at 280 g L^-1^ (Figure 3, panel B).

Glycerol has been often referred to as a “secondary metabolite” in wine fermentation due to its lower concentration in wine when compared to ethanol. However, glycerol is not “secondary” from a metabolic point of view. Synthesis of this compound plays a major role during anaerobic growth, providing precursors for the synthesis of phospholipids, protecting yeast from high osmotic pressure (caused by high sugar concentrations found in winemaking) and maintaining cell redox balance [42,43]. However, the analysis of the effect of redox balance in the fluxes towards glycerol was troublesome, mainly due to the interconnection of redox balance and nitrogen metabolism [34,36,44]. We can still hypothesize that the lower flux towards glycerol production at 16 °C could be partly explained by the higher uptake of amino acids at this temperature as described below. The uptake and metabolism of amino acids present in the medium involved a higher consumption of NADH, and thereby, lower fluxes towards glycerol formation are needed to compensate redox balance. As the glycerol present in wine is mainly produced during the first stages of fermentation, when yeast growth takes place, this lower carbon flux towards glycerol formation at 16 °C could be partly responsible for the lower concentrations of this metabolite found by the end of wine batch fermentations carried out in our laboratory when compared to 28 °C (data not shown). Supporting this finding, Gamero et al. [45] have also recently reported lower glycerol concentrations by the end of batch fermentations performed using several *S. cerevisiae* strains at 12 °C compared to 28 °C.

The C flux towards acetic acid production was also higher at 280 g L^-1^ glucose and was always accompanied by higher fluxes towards glycerol. This fact could be explained through redox balance, as the excretion of 1 mol of acetate involves the synthesis of 2 moles of NADH [37]. Additionally, higher C flux towards glycerol biosynthesis at 280 g L^-1^ glucose, irrespective of temperature, could be due to the increase in osmotic pressure.

The synthesis of lactic acid represents the remaining pathway at this node. Under anaerobic conditions, lactic acid is synthesized from dihydroxyacetone phosphate by the methylglyoxal bypass. This pathway has always been described as functional at very low rates and under ‘overflow metabolism’ conditions [32]. Our results showed small amounts of lactic acid at 28 °C (45 and 15 mg L^-1^, for 240 and 280 g L^-1^ of glucose, respectively) and concentrations below the detection limit (5 mg L^-1^) in the cultures performed at 16 °C. Low levels of lactic acid are still likely to be produced at 16 °C as all the conditions studied implied overflow metabolism through glycolysis (Figure 3, panel B).

#### Pyruvate branch point

The pyruvate node represents one of the most important regulation points in carbon metabolism and the key to energy production [32]. In this way, carbon can either follow catabolic reactions (acetaldehyde production and TCA cycle) or anabolic pathways (oxaloacetic acid production (OAA) and amino acid biosynthesis). The fate of pyruvate to branched chain amino acid (BCAA) will be thoroughly described in a following subsection.

In all cases, the C flux from pyruvate was mainly derived towards the formation of acetaldehyde, oscillating in a very narrow range from 96.4 to 97.0%. Notably, the carbon flux to TCA was reduced in about 30% at 16 °C compared to 28 °C, regardless of sugar concentration. On the other hand, the flux towards OAA was not severely affected by the variables studied (Figure 3, panel C).

Around 99% of the C flux diverted to acetaldehyde was used in the concomitant production of energy, CO_2_ and ethanol. The rest of the flux was directed to the production of acetic acid. A clear effect of sugar concentration was observed in its production, being higher at 280 g L^-1^ glucose. The increase in the flux oscillated between 20% and 30% at 16 and 28 °C, respectively (Figure 3, panel C).

In general, the metabolic flux distribution calculated at this branch point in our work agrees with that proposed by other authors under anaerobic conditions [6,23,25,40]. These similarities could indicate the tight regulation of this node under anaerobic conditions, contrary to that observed at the glucose 6-phosphate node. The slight differences found in the split acetaldehyde/TCA cycle could be related to growth rate as cells were growing faster at 28 °C and, in consequence, demanding higher amounts of building-blocks from the TCA cycle.

#### Nitrogen metabolism

Simultaneous uptake of ammonium and amino acids was observed in all of the conditions studied. Analyzing data obtained in the control condition (240 g L^-1^ glucose at 28 °C), amino acids could be differentially grouped according to the percentage of consumption in the steady states (Table S2). Thus, while the consumption of lysine accounted for 98.2% of its original amount in the feed, the consumption of several amino acids such as alanine, tryptophan or tyrosine, and ammonium was below 40%. Other amino acids such as arginine, aspartic and glutamic acid, serine, glutamine, threonine, methionine, leucine, isoleucine and phenylalanine presented intermediate consumption percentages, ranging from 40 to 80%. Our results provide additional information to previously reported data obtained using both winemaking conditions [46,47] and synthetic media with a complex mixture of nitrogen sources [36,48]. A recent study [49] has furthered the understanding of the co-consumption of organic and inorganic nitrogen sources and explained it based on the kinetic characteristics of transporters as well as nitrogen catabolite repression (NCR) and Ssy1p-Ptr3p-Ssy5 (SPS)-mediated control, and allowed the classification of the different nitrogen sources according to their order of consumption (Table S2).

When the consumption of amino acids was analyzed in the three remaining conditions, it was observed to be strongly affected both by sugar concentration, with all cases higher at 240 g L^-1^ glucose than at 280 g L^-1^ at both temperatures studied (except for tyrosine at 16 °C), and by temperature and dilution rate, with all cases higher at 28 °C than at 16 °C (Table S2). However, it is interesting to point out that the intensity of the effect exerted by these parameters clearly differed among amino acids (their consumption did not increase or decrease to the same extent). Interestingly, these differences did not seem to affect the amino acid composition of the cellular protein (Table S3) and, consequently, the C formulae of the protein determined for each condition (Table 2). The similarity in the amino acidic composition of cell proteins grown in different conditions is consistent with data reported in literature and has even been observed for *S. cerevisiae* cells grown on three different nitrogen sources [36].

Temperature and dilution rate exerted a clear effect on nitrogen metabolism while that of sugar concentration was negligible. Table 5 (panel A) shows the uptake of organic and inorganic nitrogen sources, expressed as N-mmol gDW^-1^ h^-1^. Regardless of sugar concentration, an approximate 2.4 fold increase in the total nitrogen uptake was observed at 28 °C (D = 0.25 h^-1^) compared to 16 °C (D = 0.1 h^-1^). This data is in accordance with a previous study performed in nitrogen limited anaerobic cultures of *S. cerevisiae*, which demonstrated the existence of a linear relationship between dilution rate and ammonium uptake rate [37]. However, due to the differential glucose uptake observed between conditions, the amount of nitrogen incorporated per C unit was higher at 16 °C (D = 0.1 h^-1^) than at 28 °C (Table 5, panel B) although not significantly different. In all cases, nitrogen was mostly incorporated in the form of amino acids. However, the organic/inorganic nitrogen ratio changed with growth temperature. While ammonium represented 37-38% of the total N incorporated at 28 °C and D = 0.25 h^-1^, this percentage significantly dropped to approximately 15.5-18.5% at 16 °C and D = 0.1 h^-1^ (Table 5, panel C). This result could be explained by an alleviation of NCR observed at low temperatures and deduced from the expression of genes coding for ammonium and amino acid permeases (*MEP2 and GAP1*) observed by other authors [50].

**Table 5:** Key physiological parameters related to nitrogen metabolism: uptake rate of organic and inorganic nitrogen sources expressed as N-mmol gDW^-1^ h^-1^; incorporation of total, organic and inorganic nitrogen normalized by glucose uptake (N-mmol C-mol^-1^ Glucose); percentage of organic and inorganic nitrogen incorporated; contribution of each amino acid (in percentage) to the incorporation of organic nitrogen.

	240 g L^-1^ Glucose		280 g L^-1^ Glucose	
**N-mmol gDW^-1^ h^-1^**	16 °C	28 °C	16 °C	28 °C
Total	0.70 ± 0.01^a^	1.74 ± 0.05^b^	0.70 ± 0.01^a^	1.58 ± 0.04^b^
Organic	0.57 ± 0.01^a^	1.09 ± 0.02^b^	0.59 ± 0.01^a^	0.98 ± 0.02^b^
Inorganic	0.13 ± 0.01^a^	0.64 ± 0.02^b^	0.11 ± 0.01^a^	0.60 ± 0.03^b^
**N-mmol C-mol^-1^ Glucose**				
Total	24.0 ± 0.6	19.9 ± 2.1	30.2 ± 1.5	21.5 ± 1.6
Organic	19.5 ± 0.5	12.6 ± 1.4	25.5 ± 1.3	13.3 ± 1.0
Inorganic	4.5 ± 0.3	7.4 ± 0.8	4.7 ± 0.5	8.2 ± 0.7
**% of N incorporated**				
Organic	81.3 ± 1.84^a^	63.0 ± 1.87^b^	84.4 ± 2.13^a^	61.9 ± 2.02^b^
Inorganic	18.7 ± 1.45^a^	37.0 ± 1.44^b^	15.6 ± 1.56^a^	38.1 ± 2.13^b^
**Amino acid contribution to organic N uptake (%)**				
Alanine	10.7	10.0	10.6	9.2
Arginine	36.7	41.9	37.8	42.2
Aspartate	4.9	6.2	5.5	6.7
Cysteine	0.4	1.2	0.3	1.0
Glutamine	14.3	6.6	15.1	6.3
Glutamate	2.2	3.3	1.1	3.2
Glycine	0.5	0.5	0.5	0.4
Histidine	0.3	0.1	0.1	0.2
Isoleucine	2.0	2.3	1.7	2.3
Leucine	3.6	3.7	3.5	3.8
Lysine	3.4	3.1	4.4	3.8
Methionine	1.6	1.7	1.3	1.7
Phenylalanine	1.3	1.9	1.1	1.9
Serine	8.1	7.1	7.7	7.0
Threonine	6.2	6.0	5.6	5.9
Tryptophan	1.2	1.6	1.1	1.2
Tyrosine	0.3	0.5	0.2	0.3
Valine	2.5	2.3	2.2	2.7

Different superscripts indicate statistically significant differences between values (p < 0.05).

Considering amino acids individually, it is worth mentioning that arginine, alanine and glutamine account for approximately 60% of the total organic nitrogen incorporated (approx. 40, 10 and 10%, respectively). Aspartic acid, serine and threonine represented approximately 19%, individually accounting for around 6-7%. Another group formed by glutamic acid, isoleucine, leucine, lysine, methionine, phenylalanine, tryptophan and valine accounted for around 19% of the total organic nitrogen incorporated, each of them never representing more than 4%. The individual contribution of the remaining amino acids never exceeded 1%. Detailed data are shown in Table 5, panel D.

It should be highlighted that the individual contribution of amino acids to the total nitrogen uptake differed between conditions. While the contribution of glutamine increased at low temperature, that of glutamic acid decreased (for more details, see Table 5, panel D). Overall, when the total amount of nitrogen incorporated per gram of biomass (N-mmol gDW^-1^) was analyzed, significantly higher values were obtained for the continuous cultures performed at 16 °C at both sugar concentrations. This implies that the biomass yield on nitrogen (gDW N-mmol^-1^) in a synthetic medium mimicking grape must decreases at low temperatures as had previously been reported for the industrial strain CEN.PK 113-7D and EC1118 in a medium that only presented inorganic nitrogen sources [21]. As commented in a previous section, it is important to point out that this differential consumption of organic and inorganic nitrogen sources exerts an effect on the cell redox balance and, consequently, on the production of glycerol.

These results support the data presented by other authors [50] and indicate that the quantity and quality of yeast nitrogen requirements during the exponential growth phase are not the same at optimum and low temperatures. Improved nitrogen supplementations (for, for example, white wine fermentations) could be formulated on the basis of these findings. Indeed, Martinez-Moreno et al. [51] have already demonstrated the differential effect of organic nitrogen sources on biomass formation, yeast vitality and fermentation kinetics.

#### Metabolism of fusel alcohols and their amino acidic precursors

Fusel alcohols produced by yeast during wine fermentation have a strong impact on the sensorial properties of the final product [38]. For this reason and for the first time, five of the most relevant fusel alcohols (i.e., isoamyl alcohol, active amyl alcohol, phenyl 2-ethanol, n-propanol and isobutanol) have been analyzed and included in a stoichiometric model. These compounds derive both from the catabolism of different amino acids (leucine, isoleucine, phenylalanine, threonine and valine, respectively) and/or anabolic pathways from pyruvate [43]. As discussed above, the total amount of nitrogen supplied by these five amino acids accounts for 15.6% in the conditions studied, with threonine the most prevalent (Table 5, panel D). It should be noted that the relative contribution of these amino acids to the organic fraction of nitrogen incorporated, except for phenylalanine, was very stable between all conditions studied.

The production of each of these fusel alcohols normalized to their amino acidic precursor is shown in Table 6. In general, the amount of fusel alcohol produced could be explained based on the catabolism of each of its amino acidic precursors. However, it is interesting to point out the cases of isoamyl alcohol and isobutanol production. Regardless of the condition studied, both fusel alcohols demand a higher amount of amino acidic precursors for their synthesis than that provided by the uptake from the medium. In fact, coefficients higher than 1.0 (Table 6) indicate that their synthesis could not be supported exclusively from the amino acids present in the medium (leucine and valine, respectively) at 240 g L^-1^ glucose and 28 °C. Taking into account the data obtained for the rest of the fusel alcohols included in this study and the fate of exogenous amino acids inside the cells observed by Crépin [52], we could hypothesize that the anabolic pathways involved in the synthesis of fusel alcohols are also active during the yeast exponential growth phase in all conditions studied. Additionally, this fact is supported by the results provided by our stoichiometric model.

**Table 6:** Production of fusel alcohols expressed as C-mmol per C-mmol of amino acid precursor transported into the cell.

	240 g L^-1^ Glucose		280 g L^-1^ Glucose	
	16 °C	28 °C	16 °C	28 °C
Amyl alcohol / Isoleucine	0.37	0.70	0.38	0.54
Propanol / Threonine	0.35	0.57	0.31	0.67
Isoamyl alcohol / Leucine	0.55	1.08	0.47	0.92
Isobutanol / Valine	0.37	1.86	0.42	0.83
Phenyl ethanol / Phenylalanine	0.19	0.34	0.20	0.30

The production of fusel alcohols normalized per gram of biomass (C-mmol gDW^-1^) was significantly higher in all continuous cultures performed at 28 °C (D = 0.25 h^-1^) compared to those performed at 16 °C. An effect of sugar concentration was also observed, with higher effects at 240 g L^-1^ glucose than at 280 g L^-1^ at the two temperatures assayed. These findings could also be partly explained as a result of the redox balance in the cell. As in the case of glycerol, the biosynthesis of these compounds could contribute to the regeneration of the NAD^+^.

## Conclusions

In this work, metabolic flux analysis has been applied to study the physiology of *S. cerevisiae* during the exponential growth phase in a winemaking process using two different sugar concentrations at two different fermentation temperatures. The stoichiometric model proposed in the present paper represents an extension of that described by Varela and co-workers [6] incorporating the synthesis and release of aroma compounds and represents the most complete stoichiometric model used to study yeast metabolism in winemaking thus far. Clear differences have been found in consumption and production rates of the main metabolites analyzed in the study depending mainly on temperature and dilution rate (Table S1). Despite these differences, the distribution of carbon fluxes did not change drastically when the four conditions were compared, likely due to the similarities in the stress factors employed in all cases (high sugar and low nitrogen content). However, results obtained have shown that the variables studied (temperature and sugar concentration) exerted a higher effect on the pentose phosphate pathway and glycerol formation than on glycolysis and ethanol production. On the other hand, nitrogen metabolism was strongly affected by the growth conditions studied. Specifically, clear differences in the uptake of organic and inorganic nitrogen sources were observed. These findings could be employed in the design of optimal supplementation strategies of musts containing low initial nitrogen content in order to avoid stuck of sluggish fermentations. Furthermore, the reported data support the simultaneous operation of anabolic and catabolic pathways involved in the synthesis of fusel alcohols. Additional studies are being carried out in the remaining phases of fermentation in order to contribute to the understanding of the impact of nitrogen additions on fermentation kinetics and the improvement of the aroma profile of the resulting wines.

## Supporting Information

Figure S1 PCA performed to verify the reproducibility of the steady states obtained for each condition. These two components explained 87.6% of the variance.
(TIF)

Appendix S1 Biochemical reactions included in the stoichiometric model.
(DOCX)

Table S1 Measured consumption/production rates of the different metabolites analyzed in the study. sd values correspond to the standard deviation of the average value measured in two independent biological replicates. Consumption rates are indicated with a minus sign. ND: Not detected in the analysis.
(DOC)

Table S2 Percentage of consumption of each amino acid in the steady states of each condition ((mg L^-1^ in the steady state/ mg L^-1^ in the feed)·100). Code: colour values given according to the classification proposed by Crépin et al. [49]. Pink: prematurely consumed; Green: early consumed; Blue: late consumed. Those amino acids left in white were not classified by the authors; n.d.: not determined.
(DOCX)

Table S3 Molar fraction of amino acids present in proteins. ^a^Not properly quantified in the analysis. Data from taken from Lange and Heijnen [18]. Asx: Asn + Asp; Glx: Gln + Glu.
(DOCX)
