# Supplementary figures and images for: Metabolic Flux Analysis during the Exponential Growth Phase of Saccharomyces cerevisiae in Wine Fermentations
Source: PLoS One. 2013 Aug 13;8(8):e71909. doi: 10.1371/journal.pone.0071909 (PMC3742454; doi:10.1371/journal.pone.0071909)

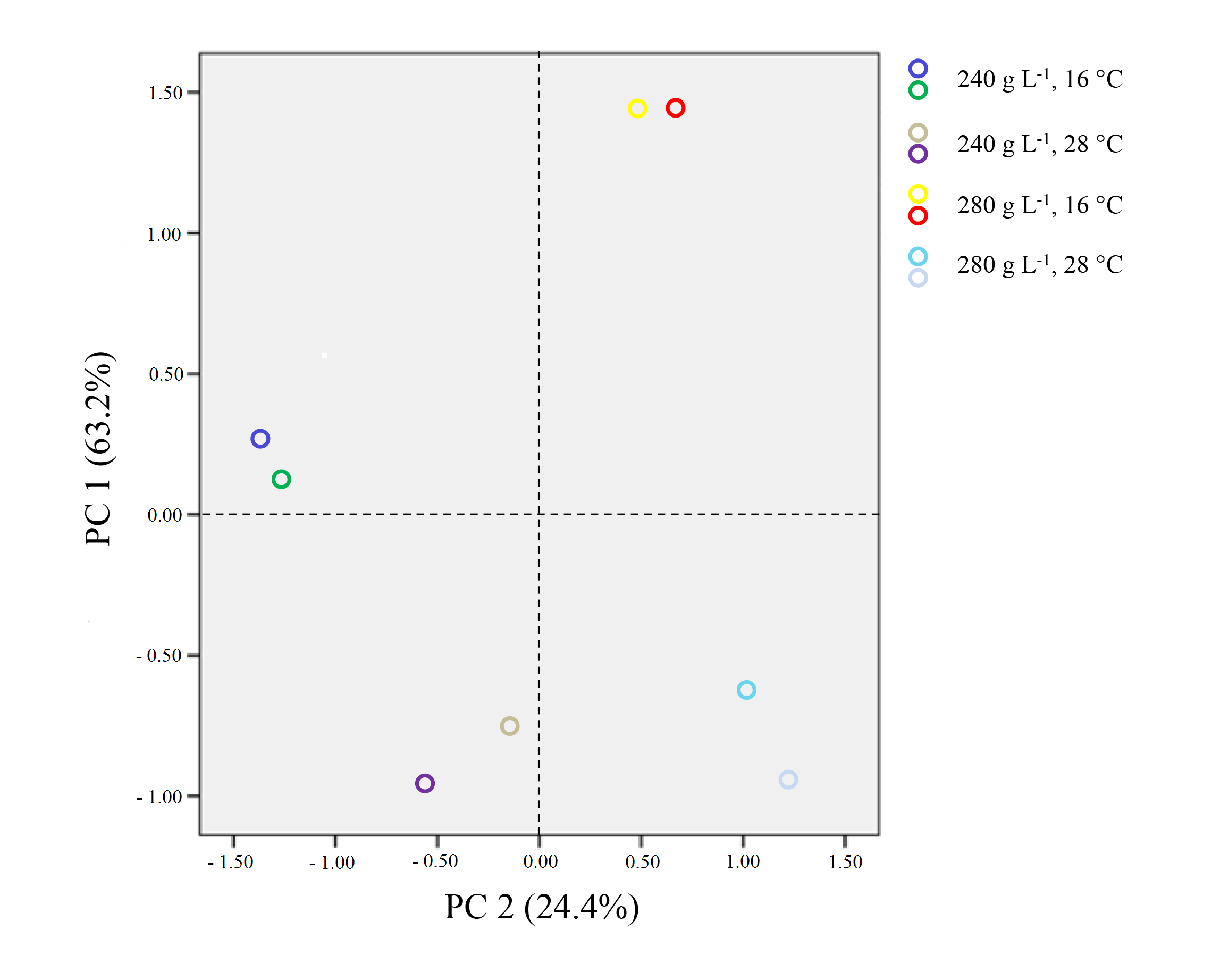

Supplement: Figure S1 — PCA performed to verify the reproducibility of the steady states obtained for each condition. These two components explained 87.6% of the variance. (TIF) [file pone.0071909.s001.tif]
