# Supplementary material for: Metabolic Flux Analysis during the Exponential Growth Phase of Saccharomyces cerevisiae in Wine Fermentations
Source: PLoS One. 2013 Aug 13;8(8):e71909. doi: 10.1371/journal.pone.0071909 (PMC3742454; doi:10.1371/journal.pone.0071909)
Supplement: Appendix S1 — Biochemical reactions included in the stoichiometric model. (DOCX) [file pone.0071909.s002.docx]

**Glycolysis**

Glc + ATP ==> G6P

G6P <==> F6P

F6P + ATP <==> DHAP + GLA3P

DHAP <==> GLA3P

GLA3P <==> 3PG + NADH + ATP

3PG <==> PEP

PEP <==> PYR + ATP

**Glycerol metabolism**

DHAP + NADH <==> GL3P

GL3P <==> GL

**Pyruvate metabolism**

PYR <==> iCO_2_ + ACAL

NADH + ACAL <==> EtOH

ACAL + NADHmit <==> EtOH

ACAL <==> NADPH + AC

AC + ATP <==> ACCOA

**Pentose phosphate pathway**

G6P <==> 2 NADPH + iCO_2_ + R5P

2 R5P <==> F6P + E4P

R5P + E4P <==> F6P + GAL3P

**Tricarboxylic acid cycle**

PYR + OAAmit <==> iCO_2_ + ICITmit + NADHmit

ICITmit <==> iCO_2_ + AKG + NADHmit

ICITmit <==> iCO_2_ + AKG + NADPHmit

AKG <==> iCO_2_ + SUCCmit + ATP + NADHmit

FUMmit <==> OAAmit + NADHmit

**Anaplerotic reaction**

PYR + iCO_2_ + ATP <==> OAA

**Amino acid metabolism**

NADPH + AKG + iNH_4_ <==> GLU

ATP + iNH_4_ + GLU <==> GLN

OAA + GLU <==> AKG + ASP

3PG + GLU <==> NADH + AKG + SER

PYR + GLU <==> AKG + ALA

2 NADPH + 2 ATP + ASP <==> THR

THR + NADH <==> iCO_2_ + PROPex

2 PYR + ACCOA + GLU <==> 2 iCO_2_ + 2 NADH + AKG + LEU

ACCOA + NADHmit + GLU + VAL <==> iCO_2_ + NADH + AKG + LEU

AKG + LEU <==> iCO_2_ + GLU + IAMI-OH

2 PYR + NADPH + GLU <==> iCO_2_ + AKG + VAL

AKG + VAL <==> iCO_2_ + GLU + IBUT-OH

2 PEP + NADPH + E4P + ATP + GLU <==> iCO_2_ + AKG + PHE

AKG + PHE <==> iCO_2_ + GLU + PHEEtOH

PYR + NADPHmit + GLU + THR <==> iCO_2_ + AKG + iNH_4_ + ILE

AKG + ILE <==> iCO_2_ + GLU + AMI-OH

2 NADPH + ACCOA + 3 ATP + 2 GLU <==> iCO_2_ + 2 NADH + AKG + LYS

2 ATP + iNH_4_ + ASP <==> ASN

2 NADPH + iCO_2_ <==> MTHF

SER <==> MTHF + GLY

3 NADPHmit + 3 ATP + ASP + MTHF + CYS <==> PYR + iNH_4_ + MET

2 PEP + NADPH + E4P + 3 ATP + GLU <==> iCO_2_ + NADH + AKG + TYR

NADPH + iCO_2_ + R5P + 5 ATP + iNH_4_ + GLN <==> 2 NADH + AKG + HIS

AKG + TRP <==> iCO_2_ + GLU + I3-EtOH

NADPH + AKG + ARG <==> iCO_2_ + 2 iNH_4_ + GLU + PRO

NADH + AC + CYS ==> 5 NADPH + ACCOA + ATP + SER

**Nitrogen uptake**

ATP + NH_4_ex ==> iNH_4_

ATP + ALAex ==> ALA

ATP + ARGex ==> ARG

ATP + ASPex ==> ASP

ATP + CYSex ==> CYS

ATP + GLNex ==> GLN

ATP + GLUex ==> GLU

ATP + GLYex ==> GLY

ATP + HISex ==> HIS

ATP + ILEex ==> ILE

ATP + LEUex ==> LEU

ATP + LYSex ==> LYS

ATP + METex ==> MET

ATP + PHEex ==> PHE

ATP + SERex ==> SER

ATP + THRex ==> THR

ATP + TRPex ==> TRP

ATP + TYRex ==> TYR

ATP + VALex ==> VAL

**Product release**

AC ==> ACex

EtOH <==> EtOHex

GL <==> GLex

SUCCmit ==> SUCCex

iCO_2_ ==> CO_2_ex

DHAP ==> LACex

**Synthesis of AICAR**

iCO_2_ + R5P + 6 ATP + 2 GLN + ASP + SER ==> NADPH + FUMmit + 2 GLU + 9 AICAR

**Maintenance**

ATP ==>

**Macromolecules biosynthesis**

*Synthesis of nucleic acid*

0.057 iCO_2_ + 0.048 R5P + 0.132 NADH + 0.489 ATP + 0.105 GLN + 0.075 ASP + 0.511 AICAR ==> 0.135 NADPH + 0.027 FUMmit + 0.105 GLU + NA

*Synthesis of carbohydrates*

G6P + ATP ==> 6 CARB

*Synthesis of lipids*

0.022 GAL3P + 0.831 NADPH + 0.416 ACCOA + 0.400 ATP + 0.034 SER ==> LIP

*Synthesis of proteins*

240 g L^-1^ glucose and 16 °C

0.015 NADPHcyt + 4 ATP + 0.087 ALA + 0.080 ARG + 0.051 ASN + 0.051 ASP + 0.004 CYS + 0.051 GLU + 0.051 GLN + 0.081 GLY + 0.024 HIS + 0.046 ILE + 0.076 LEU + 0.082 LYS + 0.020 MET + 0.035 PHE + 0.044 PRO + 0.062 SER + 0.058 THR + 0.008 TRP + 0.025 TYR + 0.063 VAL = 4.887 PROT

240 g L^-1^ glucose and 28 °C

0.032 NADPHcyt + 4 ATP + 0.092 ALA + 0.090 ARG + 0.050 ASN + 0.050 ASP + 0.004 CYS + 0.050 GLU + 0.050 GLN + 0.081 GLY + 0.022 HIS + 0.045 ILE + 0.074 LEU + 0.079 LYS + 0.021 MET + 0.034 PHE + 0.042 PRO + 0.064 SER + 0.058 THR + 0.009 TRP + 0.024 TYR + 0.062 VAL = 4.871 PROT

280 g L^-1^ glucose and 16 °C

0.0001 NADPHcyt + 4 ATP + 0.085 ALA + 0.088 ARG + 0.051 ASN + 0.051 ASP + 0.004 CYS + 0.052 GLU + 0.052 GLN + 0.080 GLY + 0.024 HIS + 0.046 ILE + 0.075 LEU + 0.080 LYS + 0.020 MET + 0.034 PHE + 0.041 PRO + 0.061 SER + 0.057 THR + 0.008 TRP + 0.026 TYR + 0.0623 VAL = 4.905 PROT

280 g L^-1^ glucose and 28 °C

0.001 NADPHcyt + 4 ATP + 0.088 ALA + 0.088 ARG + 0.050 ASN + 0.050 ASP + 0.0041 CYS + 0.051 GLU + 0.051 GLN + 0.083 GLY + 0.027 HIS + 0.045 ILE + 0.075 LEU + 0.079 LYS + 0.020 MET + 0.034 PHE + 0.041 PRO + 0.062 SER + 0.058 THR + 0.008 TRP + 0.024 TYR + 0.061 VAL = 4.883 PROT

*Synthesis of biomass*

240 g L^-1^ glucose and 16 °C

0.080 NADPHcyt + 0.290 CARB + 0.086 RNA + 0.590 PROT + 0.030 LIP = 1 BIOM

240 g L^-1^ glucose and 28 °C

0.039 NADPHcyt + 0.270 CARB + 0.145 RNA + 0.490 PROT + 0.100 LIP = 1 BIOM

280 g L^-1^ glucose and 16 °C

0.075 NADPHcyt + 0.340 CARB + 0.061 RNA + 0.590 PROT + 0.010 LIP = 1 BIOM

280 g L^-1^ glucose and 28 °C

0.036 NADPHcyt + 0.250 CARB + 0.132 RNA + 0.480 PROT + 0.130 LIP = 1 BIOM

**Abbreviations**

3PG 3-Phospho-D-glycerate

ACCOA Acetyl-CoA

ACAL Acetaldehyde

AC Acetate

ACex Extracellular Acetate

AICAR 1-(5'-Phosphoribosyl)-5-amino-4-imidazolecarboxamide

AKG 2-Oxoglutarate

ALA L-Alanine

ALAex Extracellular L-Alanine

AMI-OH Amylalcohol

ARG L-Arginine

ARGex Extracellular L-Arginine

ASN L-Asparragine

ASP L-Aspartate

ASPex Extracellular L-Aspartate

ATP Adenosin Triphosphate

BIOM Biomass

CARB Carbohydrate

CO_2_ex Extracellular CO_2_

CYS L-Cysteine

CYSex Extracellular L-Cysteine

DHAP Di-hydroxyAcetona Phosphate

E4P D-Erythrose 4-phosphate

EtOH Ethanol

EtOHex Extracellular Ethanol

F6P D-Fructose 6-phosphate

FUMmit Mitochondrial Fumarate

GLA3P Glyceraldehyde 3-Phosphate

G6P D-Glucose 6-Phosphate

GLN L-Glutamine

GLNex Extracellular L-Glutamine

GLU L-Glutamate

Glc D-Glucose

GLUex Extracellular L-Glutamate

GLY Glycine

GLYex Extracellular Glycine

GL Glycerol

GLex Extracellular Glycerol

GL3P Cytosolic Glycerol 3-Phosphate

HIS L-Histidine

HISex Extracellular L-Histidine

I3-EtOH Indole-3-ethanol

IAMI-OH Isoamyl alcohol

IBUT-OH Isobutanol

ICITmit Mitochondrial isocitrate

iCO_2_ Intracellular CO_2_

ILE L-Isoleucine

ILEex Extracellular L-Isoleucine

iNH_4_ Intracellular NH_4_

LACex Extracellular Lactate

LEU L-Leucine

LEUex Extracellular L-Leucine

LIP Lipid

LYS L-Lysine

LYSex Extracellular L-Lysine

MET L-Methionine

METex Extracellular L-Methionine

MTHF 5,10-Methyltetrahydrofolate

NADH Cytosolic NADH

NADHmit Mitochondrial NADH

NADPH Cytosolic NADPH

NADPHmit Mitochondrial NADPH

NH_4_ex Extracellular NH_4_

OAA Cytosolic Oxaloacetate

OAAmit Mitochondrial Oxaloacetate

PEP Cytosolic Phosphoenolpyruvate

PHE L-Phenylalanine

PHEEtOH 2-Phenyl ethanol

PHEex Extracellular L-Phenylalanine

PRO L-Proline

PROPex Extracellular n-Propanol

PROT Protein

PYR Pyruvate

R5P D-Ribose 5-Phosphate

NA Nucleic acid

SER L-Serine

SERex Extracellular L-Serine

SUCCex Extracellular Succinate

SUCCmit Mitochondrial Succinate

THR L-Threonine

THRex Extracellular L-Threonine

TRP L-Triptophan

TRPex Extracellular L-Triptophan

TYR L_Tyrosine

TYRex Extracellular L-Tyrosine

VAL L-Valine

VALex Extracellular L-Valine
