# Supplementary material for: Metabolic Flux Analysis during the Exponential Growth Phase of Saccharomyces cerevisiae in Wine Fermentations
Source: PLoS One. 2013 Aug 13;8(8):e71909. doi: 10.1371/journal.pone.0071909 (PMC3742454; doi:10.1371/journal.pone.0071909)
Supplement: Table S1 — Measured consumption/production rates of the different metabolites analyzed in the study. sd values correspond to the standard deviation of the average value measured in two independent biological replicates. Consumption rates are indicated with a minus sign. ND: Not detected in the analysis. (DOC) [file pone.0071909.s003.doc]

|  | 240 g L^-1^ Glucose | | | | 280 g L^-1^ Glucose | | | |
| --- | --- | --- | --- | --- | --- | --- | --- | --- |
|  | 16 °C | | 28 °C | | 16 °C | | 28 °C | |
|  | mmol gDW^-1^ h^-1^ | sd | mmol gDW^-1^ h^-1^ | sd | mmol gDW^-1^ h^-1^ | sd | mmol gDW^-1^ h^-1^ | sd |
| Glucose | -4.76 | 0.56 | -20.51 | 0.98 | -4.19 | 0.61 | -16.31 | 1.36 |
| Glycerol | 0.38 | 0.01 | 1.37 | 0.15 | 0.34 | 0.02 | 1.39 | 0.12 |
| Ethanol | 8.29 | 0.24 | 25.7 | 2.00 | 6.79 | 0.30 | 21.47 | 1.82 |
| Succinic acid | 0.02 | 0.00 | 0.04 | 0.01 | 0.01 | 0.00 | 0.03 | 0.00 |
| Acetic acid | 0.12 | 0.00 | 0.29 | 0.05 | 0.12 | 0.01 | 0.36 | 0.03 |
| Lactic acid | ND | - | 0.1157 | 0.0139 | ND | - | 0.0424 | 0.0008 |
| Ala | -0.0353 | 0.0007 | -0.0925 | 0.0101 | -0.0178 | 0.0006 | -0.0705 | 0.0037 |
| Arg | -0.0724 | 0.0013 | -0.1427 | 0.0153 | -0.0568 | 0.0025 | -0.1426 | 0.0128 |
| Asp | -0.013 | 0.0006 | -0.0335 | 0.0021 | -0.0148 | 0.0005 | -0.0322 | 0.0034 |
| Cys | ND | - | -0.0045 | 0.0015 | ND | - | -0.0032 | 0.0001 |
| Gln | -0.0852 | 0.0038 | -0.2004 | 0.0254 | -0.0614 | 0.0027 | -0.1552 | 0.0067 |
| Glu | -0.0181 | 0.0006 | -0.053 | 0.0037 | -0.0092 | 0.0001 | -0.0449 | 0.0029 |
| Gly | -0.0015 | 0.0004 | -0.0049 | 0.0005 | -0.0008 | 0.0001 | -0.0033 | 0.0007 |
| His | 0.0017 | 0.0001 | 0.0007 | 0.0021 | 0.0006 | 0.0000 | 0.0007 | 0.0028 |
| Ile | -0.0112 | 0.0002 | -0.0244 | 0.0031 | -0.0093 | 0.0003 | -0.0223 | 0.001 |
| Leu | -0.0182 | 0.0005 | -0.0392 | 0.0047 | -0.0164 | 0.0004 | -0.0359 | 0.0014 |
| Lys | -0.0095 | 0.0004 | -0.017 | 0.0022 | -0.0129 | 0.0003 | -0.0187 | 0.0004 |
| Met | -0.0087 | 0.0002 | -0.018 | 0.0017 | -0.0077 | 0.0003 | -0.0168 | 0.0001 |
| NH_4_ | -0.0657 | 0.0008 | -0.5219 | 0.0331 | -0.0555 | 0.0014 | -0.494 | 0.036 |
| Phe | -0.0066 | 0.0002 | -0.0206 | 0.0021 | -0.005 | 0.0001 | -0.0181 | 0.0005 |
| Ser | -0.0389 | 0.0012 | -0.0733 | 0.0077 | -0.0327 | 0.0013 | -0.0625 | 0.0028 |
| Thr | -0.0324 | 0.0009 | -0.0635 | 0.0076 | -0.0285 | 0.0011 | -0.0552 | 0.0022 |
| Trp | -0.0033 | 0.0012 | -0.0129 | 0.0031 | -0.0016 | 0.0001 | -0.0099 | 0.0007 |
| Tyr | -0.0003 | 0.0004 | -0.0051 | 0.0007 | -0.0006 | 0.0001 | -0.0025 | 0.0002 |
| Val | -0.0119 | 0.0009 | -0.0234 | 0.0008 | -0.0092 | 0.0003 | -0.0253 | 0.0016 |
| Amyl alcohol | 0.0024 | 0.0002 | 0.0095 | 0.001 | 0.0021 | 0.0002 | 0.0067 | 0.0005 |
| Isoamyl alcohol | 0.0062 | 0.0004 | 0.0242 | 0.0026 | 0.0053 | 0.0004 | 0.019 | 0.0015 |
| Propanol | 0.0081 | 0.0031 | 0.0248 | 0.0001 | 0.0069 | 0.0006 | 0.0253 | 0.0064 |
| Isobutanol | 0.0024 | 0.0002 | 0.0183 | 0.0016 | 0.0025 | 0.0001 | 0.0101 | 0.0003 |
| Phenyl ethanol | 0.0009 | 0.0001 | 0.0046 | 0.0002 | 0.0009 | 0.0001 | 0.0035 | 0.0002 |
| Biomass | 3.72 | 0.00 | 9.02 | 0.00 | 3.75 | 0.00 | 8.68 | 0.00 |
| CO_2_ | 9.04 | 0.16 | 25.78 | 3.11 | 6.88 | 0.33 | 21.31 | 1.23 |

**Table S1.** Measured consumption/production rates of the different metabolites analyzed in the study. sd values correspond to the standard deviation of the average value measured in two independent biological replicates. Consumption rates are indicated with a minus sign. ND: Not detected in the analysis.
