# Supplementary material for: Metabolic Flux Analysis during the Exponential Growth Phase of Saccharomyces cerevisiae in Wine Fermentations
Source: PLoS One. 2013 Aug 13;8(8):e71909. doi: 10.1371/journal.pone.0071909 (PMC3742454; doi:10.1371/journal.pone.0071909)
Supplement: Table S2 — Percentage of consumption of each amino acid in the steady states of each condition ((mg L-1 in the steady state/ mg L-1 in the feed)·100). Code: colour values given according to the classification proposed by Crépin et al. [49]. Pink: prematurely consumed; Green: early consumed; Blue: late consumed. Those amino acids left in white were not classified by the authors; n.d.: not determined. (DOCX) [file pone.0071909.s004.docx]

|  | 240 g L^-1^ Glucose | | 280 g L^-1^ Glucose | |  |
| --- | --- | --- | --- | --- | --- |
|  | 16 °C | 28 °C | 16 °C | 28 °C | Code |
| Ala | 25.2 | 37.5 | 9.4 | 25.9 |  |
| Arg | 39.8 | 44.2 | 23.1 | 40.3 |  |
| Asp | 47.3 | 61.6 | 36.4 | 52.2 |  |
| Cys | n.d. | 18.8 | n.d. | 14.6 |  |
| Gln | 37.8 | 47.3 | 20.1 | 33.4 |  |
| Glu | 24.8 | 41.1 | 9.1 | 30.9 |  |
| Gly | 7.7 | 13.5 | 2.8 | 8.5 |  |
| His | n.d. | n.d. | n.d. | n.d. |  |
| Ile | 53.9 | 66.5 | 32.8 | 55.3 |  |
| Leu | 59.4 | 72.9 | 39.7 | 61.1 |  |
| Lys | 97.1 | 98.2 | 96.6 | 97.7 |  |
| Met | 71.6 | 79.5 | 47.8 | 70.0 |  |
| NH_4_ | 8.3 | 37.3 | 5.1 | 31.2 |  |
| Phe | 35.6 | 62.7 | 19.3 | 50.3 |  |
| Pro | 0 | 0 | 0 | 0 |  |
| Ser | 61.8 | 65.8 | 38.4 | 50.7 |  |
| Thr | 60.8 | 67 | 39.2 | 52.8 |  |
| Trp | 7.4 | 15 | 2.7 | 11.5 |  |
| Tyr | 3.0 | 31.0 | 5.1 | 14.2 |  |
| Val | 38.3 | 46.3 | 22 | 41.6 |  |

**Table S2.** Percentage of consumption of each amino acid in the steady states of each condition ((mg L^-1^ in the steady state/ mg L^-1^ in the feed)·100). Code: colour values given according to the classification proposed by Crépin et al. [49]. Pink: prematurely consumed; Green: early consumed; Blue: late consumed. Those amino acids left in white were not classified by the authors; n.d.: not determined.
