# Supplementary material for: Metabolic Flux Analysis during the Exponential Growth Phase of Saccharomyces cerevisiae in Wine Fermentations
Source: PLoS One. 2013 Aug 13;8(8):e71909. doi: 10.1371/journal.pone.0071909 (PMC3742454; doi:10.1371/journal.pone.0071909)
Supplement: Table S3 — Molar fraction of amino acids present in proteins. aNot properly quantified in the analysis. Data from taken from Lange and Heijnen [18]. Asx: Asn + Asp; Glx: Gln + Glu. (DOCX) [file pone.0071909.s005.docx]

|  | 240 g L^-1^ Glucose | | 280 g L^-1^ Glucose | |
| --- | --- | --- | --- | --- |
|  | 16 °C | 28 °C | 16 °C | 28 °C |
| Ala | 0.087 | 0.092 | 0.085 | 0.088 |
| Arg | 0.080 | 0.090 | 0.088 | 0.088 |
| Asx | 0.102 | 0.100 | 0.102 | 0.100 |
| Cys^a^ | 0.004 | 0.004 | 0.004 | 0.004 |
| Glx | 0.102 | 0.100 | 0.105 | 0.101 |
| Gly | 0.081 | 0.081 | 0.080 | 0.083 |
| His | 0.024 | 0.022 | 0.024 | 0.027 |
| Ile | 0.046 | 0.045 | 0.046 | 0.045 |
| Leu | 0.076 | 0.074 | 0.075 | 0.075 |
| Lys | 0.082 | 0.079 | 0.080 | 0.079 |
| Met^a^ | 0.020 | 0.021 | 0.020 | 0.020 |
| Phe | 0.035 | 0.034 | 0.034 | 0.034 |
| Pro | 0.044 | 0.042 | 0.041 | 0.041 |
| Ser | 0.062 | 0.064 | 0.061 | 0.062 |
| Thr | 0.058 | 0.058 | 0.057 | 0.058 |
| Trp^a^ | 0.008 | 0.008 | 0.008 | 0.008 |
| Tyr | 0.025 | 0.024 | 0.026 | 0.024 |
| Val | 0.063 | 0.062 | 0.063 | 0.061 |
| **Sum** | 0.999 | 1.000 | 0.999 | 0.998 |

**Table S3.** Molar fraction of amino acids present in proteins**.** ^a^Not properly quantified in the analysis. Data from taken from Lange and Heijnen [18]. Asx: Asn + Asp; Glx: Gln + Glu.
